# A Protocol Layer Trust-Based Intrusion Detection Scheme for Wireless Sensor Networks

**DOI:** 10.3390/s17061227

**Published:** 2017-05-27

**Authors:** Jian Wang, Shuai Jiang, Abraham O. Fapojuwo

**Affiliations:** 1 School of Electronic Science and Engineering, National University of Defense Technology, Changsha 410073, China; jiangshuai93@163.com; 2 Department of Electrical and Computer Engineering, University of Calgary, Calgary, AB T2N 1N4, Canada; fapojuwo@ucalgary.ca

**Keywords:** protocol layer, intrusion detection, wireless sensor networks

## Abstract

This article proposes a protocol layer trust-based intrusion detection scheme for wireless sensor networks. Unlike existing work, the trust value of a sensor node is evaluated according to the deviations of key parameters at each protocol layer considering the attacks initiated at different protocol layers will inevitably have impacts on the parameters of the corresponding protocol layers. For simplicity, the paper mainly considers three aspects of trustworthiness, namely physical layer trust, media access control layer trust and network layer trust. The per-layer trust metrics are then combined to determine the overall trust metric of a sensor node. The performance of the proposed intrusion detection mechanism is then analyzed using the t-distribution to derive analytical results of false positive and false negative probabilities. Numerical analytical results, validated by simulation results, are presented in different attack scenarios. It is shown that the proposed protocol layer trust-based intrusion detection scheme outperforms a state-of-the-art scheme in terms of detection probability and false probability, demonstrating its usefulness for detecting cross-layer attacks.

## 1. Introduction

Wireless sensor networks (WSNs) are widely used in variety of fields, such as agricultural, environmental, industrial and military monitoring applications. WSNs can be divided into flat- architecture WSNs and clustered-architecture WSNs according to their architecture. In the flat- architecture WSNs, all the sensor nodes (SNs) transmit their own data or relay data for other nodes to the base station (BS). In the clustered-architecture WSNs, the adjacent SNs are organized as a cluster, and a cluster head (CH) controls its cluster. The SNs that belong to the same cluster can only exchange information via their CH and the CH transmits information either directly or through some other CHs to the BS. Clustering brings many advantages, such as energy efficiency, better network communication, efficient topology management, minimized delay, and so on.

WSNs face serious security problems, because of the openness of nodal deployment and wireless communication. In some WSN deployments, the SNs may be captured and the key information might be leaked or compromised. The purpose of an attacker is to disrupt the security attributes of WSNs, including confidentiality, integrity, availability and authentication. To achieve these objectives, the attacker may launch attacks from different protocol layers of WSNs. At the physical layer, the attacker can jam the physical channel by interfering with the radio frequencies that nodes use for communication [1]. The attacker can also extract the secret information from the captured node, tamper with its circuitry, modify the program codes, or even replace it with a malicious node [2]. Attacks at the medium access control (MAC) layer aim to disrupt the availability of the network by purposefully creating collisions, obtain unfair priority in the contention for the channel or dissipate the limited energy of nodes. Attacks at the MAC layer include collision, denial of sleep, Guaranteed Time Slot (GTS) attack, back-off manipulation and so on [3,4,5,6]. Attacks at the network layer aim to disrupt the network routing, and acquire or control the data flows. Examples are spoofed routing information, selective packet forwarding, sinkhole, wormhole, blackhole, sybil, and hello flood attack [7,8,9,10]. Besides the attacks aiming at a single protocol layer, there are cross-layer attacks which relate to multiple layers in WSNs [11,12,13]. Cross-layer attack can achieve better attack effects, better conceal the attack behavior or reduce the cost of attack compared to the attacks at a single layer.

Considering the limited resources of the SNs, it is not realistic for WSNs to implement high-strength security mechanisms. Furthermore, the attacker may have the ability of breaking through or bypassing the protection of security mechanism with the progress of attack technologies. Thus serving as a second wall, intrusion detection plays an important role in protecting the network. The intrusion detection system for WSNs can detect whether there are behaviors violating the security policy and record evidence of being attacked by collecting and analyzing the information from sensor nodes and networks. It can send alarm timely to the system administrator and perform some countermeasures against the attack.

There are now two kinds of intrusion detection systems [14]. One is the misuse detection system, the other is the anomaly detection system. Misuse detection is based on predefined rules, where it is easy to detect known attacks, but impossible to detect unknown attacks. Anomaly detection compares present activities with normal system status and user behaviors to detect anomalies. Compared with misuse detection, anomaly detection has higher detection rate and the ability to detect unknown attacks, with its false positive rate increasing correspondingly. The focus of this paper is on anomaly detection schemes. Recently, different types of anomaly detection schemes based on traffic prediction [15], statistical method [16], data mining [17], game theory [18,19,20], immune theory [21], or trust management [22,23,24,25,26,27,28,29,30,31,32,33,34,35,36,37], etc., have been proposed.

However, there are still some unsolved issues in the existing intrusion detection schemes for WSNs. Many of the schemes detect attacks according to the anomalies of network traffic. Actually, it is a great challenge to distinguish normal behavior from abnormal behavior because not all of attacks on WSNs will introduce abnormal network traffic. Many intrusion detection schemes only aim to detect several typical types of attacks, while the scenarios of different types of attacks carried out concurrently or cross-layer attacks are seldom considered. The attack behaviors on WSNs are usually interconnected and transformed mutually. It is difficult to obtain good detection performance by only studying how to detect a certain kind of attack. Therefore, it is necessary to pay more attention to complex attack behaviors, such as cross-layer attack, and study how to utilize the protocol feature parameters at different protocol layers, especially the key parameters which may have an important influence on the performance of the network in order to improve the detection ability of intrusion detection systems [38].

In this paper, we propose a trust-based intrusion detection scheme which uses the deviations of parameters of multiple protocol layers as trust metrics, considering that the attacks will inevitably have impacts on the parameters of the different protocol layers. Inspired by the method proposed by Bao et al. [34,35], we utilize weighting method to build the system model and t-distribution to analyze the performance of our scheme. In our scheme, the monitoring node observes the key parameters of the monitored nodes at the physical layer, MAC layer and network layer, and calculates the deviations of these key parameters. According to the deviations of the parameters, the monitoring node can evaluate the trustworthiness toward the monitored node by aggregating the trust values at different layers and send it to the CH or BS. The CH or BS can then calculate the aggregated trust value of a node according to the trust values which are evaluated by multiple monitoring nodes. If the trust value of a node is less than a predefined threshold, the node is regarded as abnormal. Because the key parameters of multiple layers are being monitored, it is effective for our scheme to detect different types of attacks at different protocol layers. Moreover, our scheme is applicable to both clustered WSNs and flat WSNs.

The rest of this paper is organized as follows. Section 2 surveys existing work on trust-based intrusion detection in WSNs. Section 3 describes our intrusion detection scheme. Section 4 analyzes the performance of our scheme by using analytical and simulation approaches, and compares its performance results with those of an existing scheme in the literature. Section 5 concludes the paper.

## 2. Related Work

Trust management is an effective method to identify malicious, selfish or compromised nodes. In recent years, research on trust management and its application to intrusion detection has received considerable attention from researchers. The current trust evaluation schemes aim to improve the detection performance, resource efficiency, robustness etc., by using fuzzy theory, probability theory and statistics, weighting method, etc. [22].

In [23,24,25], fuzzy theory is used to determine the trust degree of a sensor node. Feng et al. [23] proposed a trust evaluation algorithm named as Node Behavioral strategies Banding belief theory of the Trust Evaluation algorithm (NBBTE). In their scheme, each node firstly establishes the direct and indirect trust values of neighboring nodes by comprehensively considering various trust factors and then fuzzy set theory is used to decide the trustworthiness levels of the sensor nodes. Finally, D–S evidence theory method is adopted to obtain an integrated trust value instead of a simple weighted-average one. Wu et al. [24] put forward a trust model to detect anomaly nodes in WSNs based on fuzzy theory and evidence theory. Fuzzy theory is used to calculate the trustworthiness levels of multi-dimensional characteristics of the evaluated node and the evidence theory is applied to integrate a direct trust value for the evaluated node. Shao et al. [25] proposed a lightweight and dependable trust model for clustered wireless sensor network, in which the fuzzy degree of nearness is adopted to evaluate the reliability of the recommended trust values from the third party nodes.

In [26,27], probability distribution is used to build the trust evaluation model. Ganeriwal et al. [26] presented a distributed reputation-based framework for sensor networks. It uses a watchdog mechanism to monitor communication behaviors of neighboring nodes, represents node reputation distribution using Beta distribution and calculates the trust value according to the statistical expectation of the probability reputation distribution. Luo et al. [27] proposed a dynamic trust management scheme for WSNs. It uses a hash algorithm to generate identify labels for SNs and builds a trust-evaluating model based on beta density function.

In [28,29,30,31,32,33], trust is estimated using weighting method. Atakli et al. [28] proposed a weighted-trust evaluation based scheme to detect compromised or misbehaved nodes in WSNs by monitoring their reported data. The hierarchical network can reduce the communication overhead between sensor nodes by utilizing clustered topology. Shaikh et al. [29] presented a group-based trust management scheme for clustered WSNs. It evaluates the trust of a group of nodes in contrast to traditional trust schemes that usually focus on the trust values of individual nodes, which reduces the cost of trust evaluation. Yao et al. [30] put forward a parameterized and localized trust management scheme for WSNs, where each sensor node maintains highly abstracted parameters, rates the trustworthiness of its interested neighbors to adopt appropriate cryptographic methods, identify the malicious nodes, and share the opinion locally. Li et al. [31] proposed a lightweight and dependable trust system for clustered WSNs. Given the cancellation of feedback between nodes, it can greatly improve system efficiency while reducing the effect of malicious nodes. By adopting a dependability-enhanced trust evaluating approach for cooperation between CHs, it can effectively detect and prevent malicious, selfish and faulty CHs. Jiang et al. [32] presented an efficient distributed trust model for WSNs. In their model, the trustworthiness of a node includes direct trust and indirect trust. During the calculation of direct trust, communication trust, energy trust and data trust are considered. When a subject node cannot directly observe object nodal communication behaviors, the indirect trust value is gained based on the recommendations from some other nodes. Ishmanov et al. [33] put forward a lightweight and robust trust establishment scheme using the weight of misbehavior. In their scheme, a new trust component, misbehavior frequency is introduced to improve the resiliency of the trust mechanism.

Bao et al. [34,35] utilizes weighting method to build the trust evaluation model and statistical method to analyze the false alarm probability. In [34], they presented a trust-based intrusion detection scheme using a highly scalable cluster-based hierarchical trust management protocol. It considers both quality of service trust and social trust as trust metrics and uses an analytical model based on stochastic Petri nets to evaluate the performance of the scheme, as well as a statistical method to calculate the false alarm probability. They adopt honesty to measure social trust and energy and cooperativeness to measure quality of service trust. In [35], intimacy, honesty, energy, and unselfishness are considered as four different trust components.

In [36,37], some new models are used to evaluate the trustworthiness. Zhang et al. [36] put forward a trust evaluation method for clustered wireless sensor networks based on cloud model, which implements the conversion between qualitative and quantitative of trust metrics and produces different types of trust cloud to evaluate trust values of cluster heads and cluster members. Rajeshkumar et al. [37] presented a trust based adaptive acknowledgment intrusion detection system for WSNs based on number of active successful deliveries, and Kalman filter to predict node trust.

It is important for a trust management scheme to select proper trust factors to evaluate the trustworthiness of a SN. From the literature on this topic, we can find that the trust factors of a SN is mainly based on the nodal communication behavior, energy level, or recommendation from the third party and there is no unified standard in the selection of the trust factors. The attacks initiated at each protocol layer and their influence on the parameters of the corresponding protocol layers lack comprehensive analysis. To the best of our knowledge, there is still no trust management scheme which elaborately describes the trustworthiness of a SN from the standpoint of protocol layer. Thus, it is interesting to build the trust evaluation model based on the protocol layer trust. In view of the reality of intrusion detection scheme, we mainly consider the direct trust of a node in our scheme and the trustworthiness of a node is evaluated according to its behaviors at different protocol layers. The consideration of trust worthiness from the viewpoint of multiple protocol layers distinguishes this paper from the previous related works [23,24,25,26,27,28,29,30,31,32,33,34,35,36,37]. Since the deviations of the key parameters of multiple layers are used to evaluate the trustworthiness of a node, it is helpful for our scheme to detect nodal malicious behaviors initiated from different protocol layers, which is effective for detecting cross-layer attacks.

## 3. System Model

**Figure 1:**
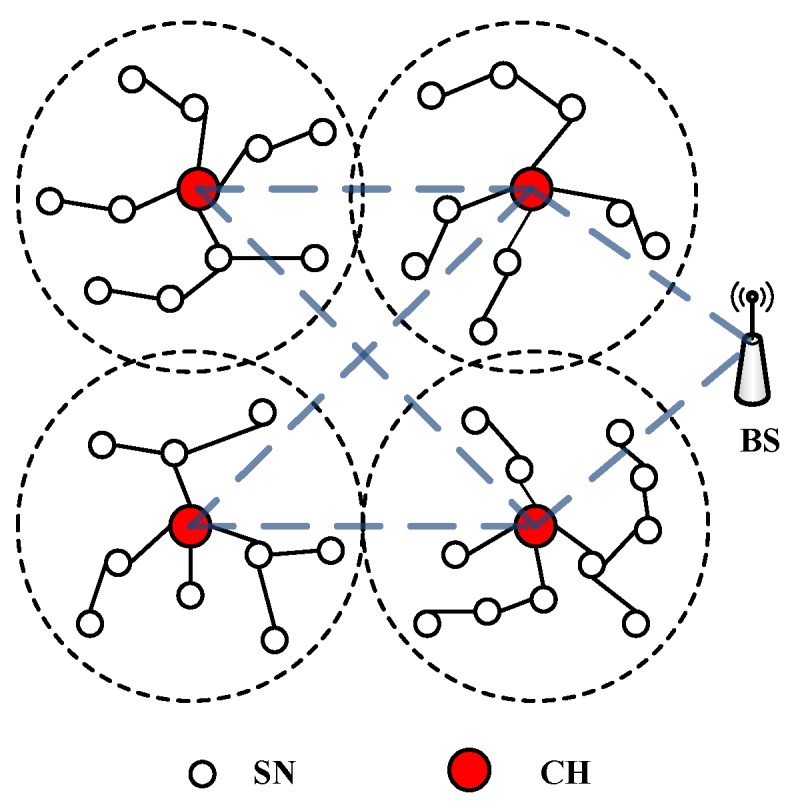
Clustered wireless sensor networks (WSN) architecture. (SN = sensor node, CH = cluster head, BS = base station).

We consider a WSN where the network can be divided into multiple clusters, as illustrated in Figure 1. Each cluster consists of a number of SNs and a CH. SNs can communicate with their CH either directly or through other SNs. A CH can forward the aggregated data to the BS directly or through other CHs.

Our trust-based intrusion detection scheme includes two levels of trust evaluation, one is CH-to-SN trust evaluation, the other is BS-to-CH trust evaluation. In CH-to-SN trust evaluation, each SN evaluates its neighbors and sends the trust evaluation results to its CH periodically. The CH evaluates all the SNs in its cluster by analyzing statistically the trust evaluation results reported by other SNs. The trust update period is Δt, which is a system parameter. The length of Δt could be made shorter or longer based on network analysis scenarios. Similarly, in BS-to-CH trust evaluation, each CH performs trust evaluation toward its neighboring CHs and sends its trust evaluation results to the BS. The BS evaluates all the CHs in the network by using the same methods as adopted in CH-to-SN trust evaluation. Since the two levels of trust evaluation use the same method, we mainly describe CH-to-SN trust evaluation.

The nodal trustworthiness consists of the trust degree of each protocol layer, including physical layer, MAC layer, network layer, transport layer and application layer. Since most of the attacks against WSNs aim at the physical layer, MAC layer and network layer, for simplicity, in this paper we mainly focus on the trusts at these three layers. Let $T_{\mathrm{ij}}^{\mathrm{DIRECT}}\left(t\right)$ denote the trust value that the sensor node i directly evaluates toward its neighboring node j at time t. It can be calculated by:

(1) $$T_{\mathrm{ij}}^{\mathrm{DIRECT}}\left(t\right)=w_{1}T_{\mathrm{ij}}^{\mathrm{PHY}}\left(t\right)+w_{2}T_{\mathrm{ij}}^{\mathrm{MAC}}\left(t\right)+w_{3}T_{\mathrm{ij}}^{\mathrm{NET}}\left(t\right),$$

where $T_{\mathrm{ij}}^{\mathrm{PHY}}\left(t\right)$, $T_{\mathrm{ij}}^{\mathrm{MAC}}\left(t\right)$, and $T_{\mathrm{ij}}^{\mathrm{NET}}\left(t\right)$ represents the trust value that node i evaluates toward node j at the physical (PHY) layer, medium access control (MAC) layer, and network (NET) layer, respectively, $w_{1}$, $w_{2}$, and $w_{3}$ are the corresponding weight values associated with these three trust components, $w_{1}\in \left[0,1\right]$, $w_{2}\in \left[0,1\right]$, $w_{3}\in \left[0,1\right]$ and $w_{1}+w_{2}+w_{3}=1$. The values of the weights $w_{1}$, $w_{2}$, and $w_{3}$ are determined according to the concrete requirement of a detection system under implementation. Generally speaking, the number of attacks aiming at the network layer is greater than those aiming at the MAC layer and physical layer. Hence, the value of $w_{3}$ is usually slightly larger than that of $w_{1}$ or $w_{2}$. In order to evaluate the trustworthiness of each protocol layer, we can choose some important parameters at each protocol layer and calculate the deviations of these parameters. Actually, our scheme is scalable. If a more accurate trust value is needed, we can choose additional parameters at each protocol layer and calculate the deviations of these parameters. Certainly, the more parameters are selected, the more complex the detection system will be. Hence, we can select parameters according to the requirement and complexity of the detection system.

The trustworthiness of a SN (or CH) should be updated periodically. Node i evaluates the trust of node j during a time window of length Δt, so the updated trust of node i toward node j is:

(2) $$T_{\mathrm{ij}}\left(t\right)={\alpha T}_{\mathrm{ij}}\left(t-\Delta t\right)+\left(1-\alpha \right)T_{\mathrm{ij}}^{\mathrm{DIRECT}}\left(t\right),$$

where $T_{\mathrm{ij}}\left(t-\Delta t\right)$ denotes the historical trust value of node i toward node j, and α∈[0,1] is the weight value of the historical trust value. Actually, the direct observation result is more important and accurate than the historical trust value. Therefore, α can be defined as $e^{-\Delta t}$. Next, we will describe the calculation of the trust at each protocol layer.

### 3.1. Calculation of Physical Layer Trust

Energy consumption rate is an important parameter at the physical layer. A malicious node usually sends or receives more packets than a normal node. It will inevitably consume more node energy, so we choose energy consumption as trust metric at this layer. The monitoring node i can obtain the energy consumption of its neighboring node j during the time period of Δt. The relative deviation of energy consumption of node j can be calculated by:

(3) $${\mathrm{RD}}_{\mathrm{EC}}\left(t\right)=\frac{\Delta E_{j}\left(t\right)-\bar{\Delta E\left(t\right)}}{\bar{\Delta E\left(t\right)}},$$

in which $\Delta E_{j}\left(t\right)=E_{j}\left(t-\Delta t\right)-E_{j}\left(t\right)$, $\bar{\Delta E\left(t\right)}=\frac{1}{n}\sum_{i=1}^{n}\Delta E_{i}\left(t\right)$.${\;E}_{j}\left(t\right)$ indicates the residual energy of node j at time t and$\;\Delta E_{j}\left(t\right)$ represents the energy consumption of node j during the time period of Δt. $\bar{\Delta E\left(t\right)}$ is the average energy consumption level of all neighboring nodes of node i during this time period and n denotes the number of neighboring nodes of node i. Node i can roughly evaluate the energy consumption of its neighboring nodes during the time period of Δt by monitoring their packet transmission activities. The greater the deviation of energy consumption is, the lower the nodal trustworthiness will be. So we obtain the physical layer trust as:

(4) $$T_{\mathrm{ij}}^{\mathrm{PHY}}\left(t\right)=\{\begin{array}{c} 1-{\mathrm{RD}}_{\mathrm{EC}}\left(t\right),\;\mathrm{if}\;0<{\mathrm{RD}}_{\mathrm{EC}}\left(t\right)<1\\ 0,\;{\mathrm{RD}}_{\mathrm{EC}}\left(t\right)\geq 1\\ 1,\;{\mathrm{RD}}_{\mathrm{EC}}\left(t\right)\leq 0 \end{array},$$

In Equation (4), if the relative deviation of energy consumption is less than or equal to 0, which means the energy consumption of the monitored node is less than the average energy consumption, the monitored node is considered trustworthy at the physical layer. If ${\mathrm{RD}}_{\mathrm{EC}}$ is greater than or equal to 1, which means the energy consumption of the monitored node is more than double or double the average energy consumption, the monitored node will be considered untrustworthy at the physical layer.

### 3.2. Calculation of MAC Layer Trust

Next we calculate the MAC layer trust. There are variety of attacks initiated at this layer whose main objective is to get the priority of channel access. A malicious node can select a small back-off time, choose a small size of contention window (CW), or wait for shorter interval than distributed inter-frame spacing (DIFS), aiming to gain significant advantage in the contention of channel over the unmalicious nodes. Therefore, the interval time between two consecutive successful transmissions of malicious node, which we define as idle time, will be less than that of the unmalicious node. The malicious node can also scramble the frames sent by other nodes in order to obtain the priority of channel access. As a result, the average number of retransmissions of the malicious node will be less than that of the unmalicious node. As described above, we choose two important parameters, the idle time and number of retransmissions, as the trust metrics at the MAC layer. Thus, at the MAC layer, the node i evaluates the trust value of node j as:

(5) $$T_{\mathrm{ij}}^{\mathrm{MAC}}\left(t\right)=p_{1}T_{\mathrm{ij}}^{\mathrm{idle}\_\mathrm{time}}\left(t\right)+p_{2}T_{\mathrm{ij}}^{\mathrm{num}\_\mathrm{retr}}\left(t\right),$$

where $p_{1}$, $p_{2}$ are the weight values associated with the two trust components, $p_{1}\in \left[0,1\right]$, $p_{2}\in \left[0,1\right]$, and $p_{1}+p_{2}=1$. The exact values of $p_{1}$ and $p_{2}$ depend on the requirements of the detection system under implementation.

**Figure 2:**
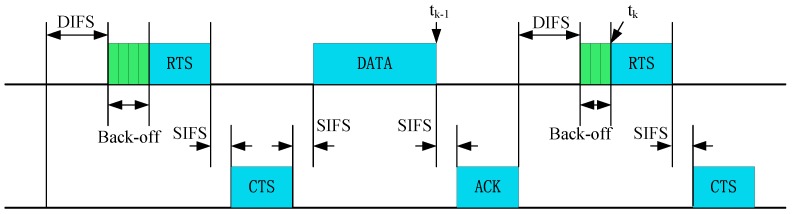
802.11 Distributed Coordination Function (DCF) operation. RTS = Request to Send, ACK = Acknowledgement, CTS = Clear To Send, DIFS= distributed inter-frame spacing, SIFS = short inter-frame spacing.

In order to calculate $T_{\mathrm{ij}}^{\mathrm{idle}\_\mathrm{time}}\left(t\right)$, the monitoring node i can obtain the idle time $x_{k}\;$ (k means the k-th transmission of the monitored node) according to Request To Send (RTS)/Clear To Send (CTS) access in Distributed Coordination Function (DCF) mode, and $x_{k}$ can be calculated by:

(6) $$x_{k}=t_{k}-t_{k-1}-t_{\mathrm{SIFS}}-t_{\mathrm{ACK}},$$

where $t_{k}$ denotes the time of the k-th RTS packet reception, $t_{k-1}$ is the end time point of the reception of the previous data segment, $t_{\mathrm{SIFS}}$ is the duration of the Short Inter-Frame Spacing (SIFS) frame, and $t_{\mathrm{ACK}}$ is the duration of Acknowledgement (ACK) frame, as illustrated in Figure 2.

For an unmalicious node, $x_{k}=t_{\mathrm{DIFS}}+b_{k}$, where $t_{\mathrm{DIFS}}$ is the duration of DIFS frame, and $b_{k}$ is the random back-off time. A malicious node is trying to decrease the idle time by manipulating the back-off time and DIFS period. Therefore, the monitoring node can detect these misbehaviors by calculating the deviation of the idle time. We can obtain the average idle time of the CH, according to:

(7) $$\bar{x}=t_{\mathrm{DIFS}}+\bar{b}=t_{\mathrm{DIFS}}+\frac{1}{u}\sum_{k=1}^{u}b_{k},$$

where u denotes the number of successful transmissions by the CH during the observation period of Δt. We then calculate the deviation of the idle time:

(8) $$D_{\mathrm{idle}\_\mathrm{time}}\left(t\right)=\frac{1}{m}\sum_{k=1}^{m}(x_{k}-\bar{x}),$$

where m is the observed number of successful transmissions of the monitored node. Therefore, the relative deviation of the idle time can be expressed as:

(9) $${\mathrm{RD}}_{\mathrm{idle}\_\mathrm{time}}\left(t\right)=\frac{\left|D_{\mathrm{idle}\_\mathrm{time}}\left(t\right)\right|}{\bar{x}}$$

and the idle time trust is calculated by:

(10) $$T_{\mathrm{ij}}^{\mathrm{idle}\_\mathrm{time}}\left(t\right)=\{\begin{array}{c} 1-{\mathrm{RD}}_{\mathrm{idle}\_\mathrm{time}}\left(t\right),\;\mathrm{if}\;\sum_{k=1}^{m}\left(x_{k}-\bar{x}\right)<0\\ 1,\;\mathrm{else} \end{array}.$$

It means that the trust value of the monitored node will decrease if its idle time is less than the average idle time.

In order to calculate the number of retransmissions trust $T_{\mathrm{ij}}^{\mathrm{num}\_\mathrm{retr}}\left(t\right)$, we first calculate the deviation of the number of retransmissions of the monitored node j. The monitoring node i can detect a retransmission by observing a repeated sequence number in the head of frames. It monitors the number of retransmissions of node j during the time period of Δt, which is denoted by $y_{\mathrm{ij}}\left(t\right)$. It can also obtain the average number of retransmissions $\bar{y\left(t\right)}$ during the time period of Δt, by monitoring the number of retransmissions of its neighboring nodes. $\bar{y\left(t\right)}=\frac{1}{n}\sum_{k=1}^{n}y_{\mathrm{ik}}\left(t\right)$, where $y_{\mathrm{ik}}\left(t\right)$ means the number of retransmissions of node k during the time period of Δt, node k is one of the neighboring nodes of node i and n denotes the number of neighboring nodes of node i. Then, the relative deviation of the number of retransmissions of node j can be calculated by:

(11) $${\mathrm{RD}}_{{\mathrm{num}}_{\mathrm{retr}}}\left(t\right)=\frac{\bar{y\left(t\right)}-y_{\mathrm{ij}}\left(t\right)}{\bar{y\left(t\right)}}$$

and the number of retransmissions trust can be expressed as:

(12) $$T_{\mathrm{ij}}^{\mathrm{num}\_\mathrm{retr}}\left(t\right)=\{\begin{array}{c} 1-{\mathrm{RD}}_{{\mathrm{num}}_{\mathrm{retr}}}\left(t\right),\;\mathrm{if}\;y_{\mathrm{ij}}\left(t\right)<\bar{y\left(t\right)}\\ 1,\;\mathrm{else} \end{array}.$$

If the number of retransmissions of node j is less than the average number of retransmissions, its trust value will decrease.

### 3.3. Calculation of Network Layer Trust

Attacks at the network layer aim to disrupt the network routing, and acquire or control the data flows. A malicious node can make itself a part of a routing path by advertising bogus routing messages, such as a good Link Quality Indicator (LQI) or a small hop count. It can also initiate sinkhole or selective forwarding attack and result in dropping all or part of forwarding packets. Therefore, we choose route metric and packet forwarding rate as trust metrics to evaluate the network layer trust. The network layer trust is described as:

(13) $$T_{\mathrm{ij}}^{\mathrm{NET}}\left(t\right)=q_{1}T_{\mathrm{ij}}^{\mathrm{route}\_\mathrm{metric}}\left(t\right)+q_{2}T_{\mathrm{ij}}^{\mathrm{PFR}}\left(t\right),$$

where $q_{1}\in \left[0,1\right]$, $q_{2}\in \left[0,1\right]$ are weight values and $q_{1}+q_{2}=1$. The exact values of $q_{1}$ and $q_{2}$ depend on the requirements of the detection system under implementation.

There are different route metrics for routing protocols in WSNs. For example, in the MintRoute protocol, it uses link estimates as routing metric and includes the LQI within its route update packet [39]. In the TinyAODV (Tiny Ad-hoc On-Demand Vector) protocol, the routing metric is the number of hop count and includes the hop count in Route Reply (RREP) packet [40]. A malicious node can make its neighbors change their current parents and choose it as their new one by advertising an attractive LQI for itself in the route update packet or giving a small value of hop count in RREP packet. We then take LQI and hop count as basis to calculate the route metric trust.

We can calculate the deviation of LQI by comparing the actual LQI value with the advertised one. When a monitoring node receives a route update packet from a monitored node, it can calculate the actual LQI value according to ${\mathrm{LQI}}_{k}^{\prime }=255\times \left({\mathrm{RSSI}}_{k}+81\right)/91$ [41], where k denotes the k-th route update packet that it received and ${\mathrm{RSSI}}_{k}$ represents the received signal strength indicator of the k-th route update packet. The monitoring node can obtain the advertised LQI from the route update packet which is denoted by ${\mathrm{LQI}}_{k}$. Then the average deviation of LQI is calculated by:

(14) $$D_{\mathrm{LQI}}\left(t\right)=\frac{1}{m}\sum_{k=1}^{m}\left({\mathrm{LQI}}_{k}\left(t-\Delta t,\;t\right)-{\mathrm{LQI}}_{k}^{\prime }\left(t-\Delta t,\;t\right)\right),$$

where m denotes the number of route update packets that the monitoring node has received during the time period of ∆t. Therefore, the LQI trust that node i evaluates toward node j can be described as:

(15) $$T_{\mathrm{ij}}^{\mathrm{LQI}}\left(t\right)=\{\begin{array}{c} 1-\frac{D_{\mathrm{LQI}}\left(t\right)}{{\mathrm{LQI}}_{\max}},\;\mathrm{if}\;\sum_{k=1}^{m}\left({\mathrm{LQI}}_{k}-{\mathrm{LQI}}_{k}^{\prime }\right)>0\\ 1,\;\mathrm{else} \end{array},$$

where ${\mathrm{LQI}}_{\max}$ equals to 255 in MintRoute protocol [39]. This formula means that the trust degree of the monitored node will decrease if the advertised LQI value is larger than the actual one.

If the route metric is hop count, the monitoring node can also evaluate the trust degree of the monitored node by calculating the deviation of hop count. The monitoring node can calculate the average hop count toward destination node according to the RREP packets it has received during the time period of Δt. The average hop count is described as $\bar{\mathrm{hop}\_\mathrm{count}}=\frac{1}{n}\sum_{k=1}^{n}\mathrm{hop}\_{\mathrm{count}}_{k}$, where n denotes the number of received RREP packets during the observation time and $\mathrm{hop}\_{\mathrm{count}}_{k}$ is the value of hop count to the destination node, which is included in the k-th RREP packet. We can also adopt the method in [42]. Each node builds a node neighbor database which contains the ID of the neighboring node and the hop count to the CH for each node. Thus, we can also calculate the average hop count by $\bar{\mathrm{hop}\_\mathrm{count}}=\frac{1}{n}\sum_{k=1}^{n}\mathrm{hop}\_{\mathrm{count}}_{k}$, where n denotes the number of neighboring nodes. The relative deviation of hop count of the monitored node j can be calculated by:

(16) $${\mathrm{RD}}_{{\mathrm{hop}}_{\mathrm{count}}}\left(t\right)=\frac{\bar{\mathrm{hop}\_\mathrm{count}}-\mathrm{hop}\_{\mathrm{count}}_{j}}{\bar{\mathrm{hop}\_\mathrm{count}}},$$

where $\mathrm{hop}\_{\mathrm{count}}_{j}$ denotes the value of hop count from node j to its CH. The hop count trust is described as:

(17) $$T_{\mathrm{ij}}^{\mathrm{hop}\_\mathrm{count}}\left(t\right)=\{\begin{array}{c} 1-{\mathrm{RD}}_{{\mathrm{hop}}_{\mathrm{count}}}\left(t\right),\;\mathrm{if}\;{\mathrm{hop}}_{\mathrm{count}}{}_{j}<\bar{\mathrm{hop}\_\mathrm{count}}\\ 1,\;\mathrm{else} \end{array}$$

This means if $\mathrm{hop}\_{\mathrm{count}}_{j}$ is less than the average hop count, the more deviation there is, and the lower the trust value will be.

In order to obtain the packet forward trust, the monitoring node i can obtain the packet forwarding rate of the monitored node j by:

(18) $$T_{\mathrm{ij}}^{\mathrm{PFR}}\left(t\right)=\frac{P_{j\to k}\left(t\right)}{P_{i\to j\to k}\left(t\right)},$$

where $P_{i\to j\to k}\left(t\right)$ denotes the number of packets that node i wants to transmit to node k with the help of node j and $P_{j\to k}\left(t\right)$ indicates the number of packets that node j has received from node i and forwarded to node k. If node j does not forward packets correctly, its trust degree will decrease.

In order to decide whether or not a node is considered compromised, it is necessary to select a system trust threshold, ${\mathrm{Th}}_{\mathrm{trust}}$. In a cluster, all of the monitoring nodes will send their trust evaluation results with respect to their neighboring nodes to their CH. The CH then computes the trust value of node j according to:

(19) $$T_{\mathrm{cj}}\left(t\right)=\frac{1}{n}\sum_{i=1}^{n}T_{\mathrm{ij}}\left(t\right),$$

where n denotes the number of neighboring nodes of node j and makes decision by comparing the trust value with ${\mathrm{Th}}_{\mathrm{trust}}$. If $T_{\mathrm{cj}}\left(t\right)$ is less than ${\mathrm{Th}}_{\mathrm{trust}}$, then node j is regarded as compromised. The method of BS-to-CH trust evaluation is similar to that of CH-to-SN trust evaluation.

## 4. Performance Analysis

The purpose of the analysis is to derive mathematical results of the false positive and false negative probabilities. The false positive probability is the probability that a node is evaluated as compromised whereas it is not. On the other hand, false negative probability is the probability that a node is evaluated as not compromised whereas it is. The expressions for the false positive and false negative probabilities are derived using a statistical approach. We also calculate the communication overhead of our scheme.

### 4.1. Statistical Analysis

We utilize t-distribution to analyze the performance of our trust-based intrusion detection scheme because it is suitable to detect the difference between two means in the circumstance of limited samples, which is similar to [34]. $T_{\mathrm{cj}}\left(t\right)$ is a random variable with normal distribution and the standard deviation of $T_{\mathrm{cj}}\left(t\right)$ is unknown. In order to calculate the false positive and false negative probabilities, we then transform $T_{\mathrm{cj}}\left(t\right)$ into a random variable $X_{j}\left(t\right)$ following t-distribution with n-1 degrees of freedom, which is denoted by:

(20) $$X_{j}\left(t\right)=\frac{T_{\mathrm{cj}}\left(t\right)-\mu_{j}\left(t\right)}{S_{j}\left(t\right)/\sqrt{n}},$$

where $T_{\mathrm{cj}}\left(t\right)=\frac{1}{n}\sum_{i=1}^{n}T_{\mathrm{ij}}\left(t\right)$ is the sample mean, $\mu_{j}\left(t\right)$ is the population mean of the trust value of node j, $S_{j}\left(t\right)=\sqrt{\frac{1}{n-1}\sum_{i=1}^{n}{\left(T_{\mathrm{ij}}\left(t\right)-T_{\mathrm{cj}}\left(t\right)\right)}^{2}}$ is the standard deviation of the trust value that node i evaluated with respect to node j, and n is the number of neighboring nodes of node j. We can obtain $\mu_{j}\left(t\right)$ by running simulations for many times. Thus, according to Equation (20), the probability that node j is evaluated as a compromised node is given by:

(21) $$P(\mu_{j}\left(t\right)<{\mathrm{Th}}_{\mathrm{trust}})=P\left(X_{j}\left(t\right)>\frac{T_{\mathrm{cj}}\left(t\right)-{\mathrm{Th}}_{\mathrm{trust}}}{S_{j}\left(t\right)/\sqrt{n}}\right).$$

The false positive probability can be calculated by:

(22) $$\rho^{\mathrm{fp}}\left(t\right)=P(X_{j}\left(t\right)>\frac{T_{\mathrm{cj}}^{N}\left(t\right)-{\mathrm{Th}}_{\mathrm{trust}}}{S_{j}^{N}\left(t\right)/\sqrt{n}}=\gamma )=\int_{\gamma }^{\infty }\frac{\Gamma \left(\frac{n+1}{2}\right)}{\sqrt{n\pi }\Gamma \left(\frac{n}{2}\right)}{\left(1+\frac{x^{2}}{n}\right)}^{-\frac{n+1}{2}}\mathrm{dx},$$

where $T_{\mathrm{cj}}^{N}\left(t\right)$ ($S_{j}^{N}\left(t\right)$) is the mean value (standard deviation) under the condition that node j is not compromised, superscript N denotes Not compromised and $\Gamma \left(x\right)=\int_{0}^{\infty }t^{x-1}e^{-t}\;\mathrm{dt}$ is the gamma function.

The false negative probability is expressed as:

(23) $$\rho^{\mathrm{fn}}\left(t\right)=P\left(X_{j}\left(t\right)\leq \frac{T_{\mathrm{cj}}^{C}\left(t\right)-{\mathrm{Th}}_{\mathrm{trust}}}{S_{j}^{C}\left(t\right)/\sqrt{n}}=\delta \right)=\int_{-\infty }^{\delta }\frac{\Gamma \left(\frac{n+1}{2}\right)}{\sqrt{n\pi }\Gamma \left(\frac{n}{2}\right)}{\left(1+\frac{x^{2}}{n}\right)}^{-\frac{n+1}{2}}\mathrm{dx},$$

where $T_{\mathrm{cj}}^{C}\left(t\right)$ ($S_{j}^{C}\left(t\right)$) is the mean value (standard deviation) under the condition that node j is compromised, superscript C denotes Compromised.

### 4.2. Numerical Results and Discussion

We use Matlab as simulation tool to generate the performance results of our scheme. We consider a WSN with 50 nodes, randomly deployed in a 100 m × 100 m operational area. The transmitting power of a SN is 2 mW and the communication frequency is 2.4 GHz. The trust update interval is set to 10–100 min. The detailed simulation parameters are listed in Table 1.

**Figure 3:**
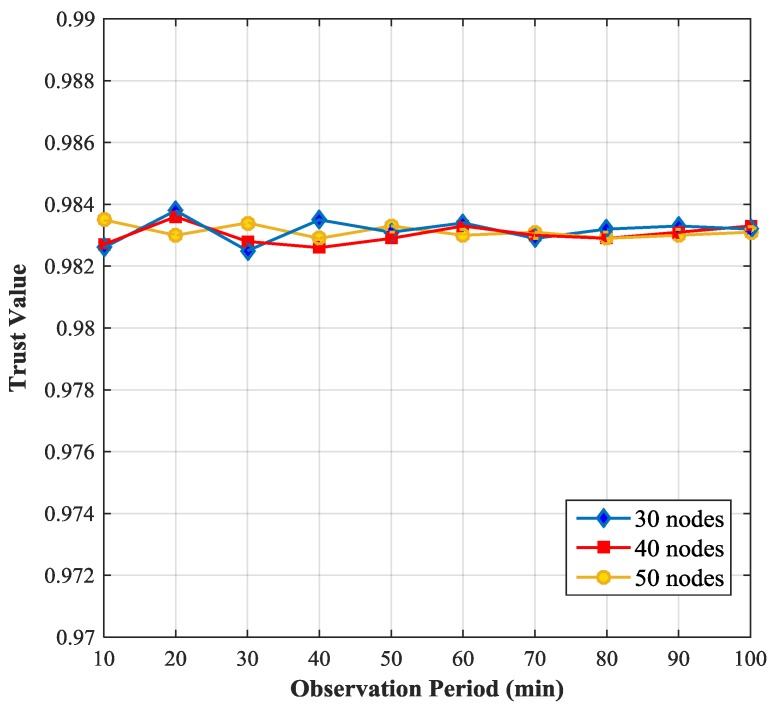
Sensitivity of trust value to simulation time and node density.

Figure 3 shows the relationship between the trust value of a SN and the simulation time and compares the trust value of the SN with the node density varying from 30 nodes to 50 nodes per 10,000 m^2^ (e.g., 30 nodes mean 1 CH and 29 SNs). We observe that the trust value of the SN fluctuates in a narrow range (0.982, 0.984), when the simulation time is relatively short. If the simulation time is long enough, the trust value of the SN becomes stable, because the longer the simulation time is, the more data are collected and the more accurate the results are. We also notice that with the increase of node density the fluctuation of the trust value of the monitored node becomes smaller. This is because if the node density is small, the number of neighboring nodes of the monitored node will be small, the data that the monitoring node can obtain will be less and hence the trust value of the monitored node will not be so accurate.

**Figure 4:**
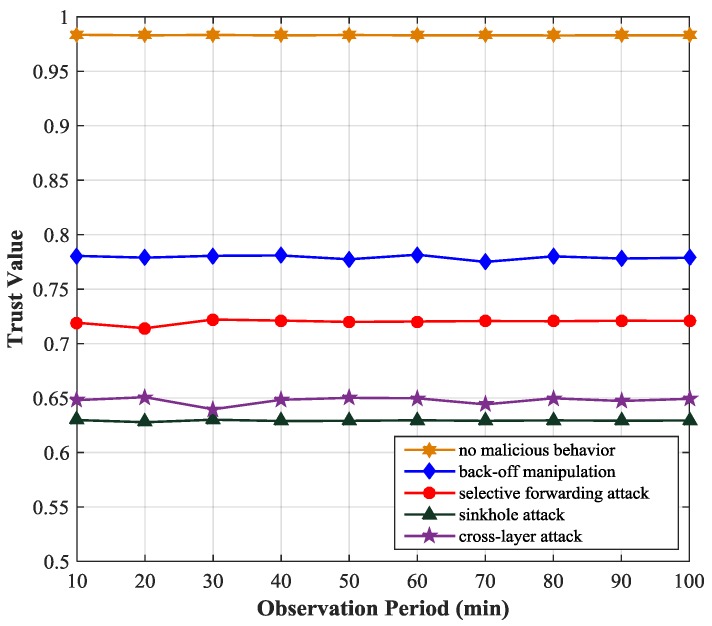
Trust values under different kinds of attacks.

Figure 4 shows the variation of the trust values of the monitored node under the scenario of several types of attacks. We simulate four typical attacks at the MAC layer and network layer, including back-off manipulation, selective forwarding attack, sinkhole attack and MAC-Network cross-layer attack. In the back-off manipulation attack, a malicious node gets unfair priority access to the channel by setting a small CW. In the selective forwarding attack, a malicious node selectively drops packets passing though it according to a predefined criterion. In the sinkhole attack, an attacker tries to attract network traffic by sending bogus RREP messages. In the MAC-Network cross-layer attack, the malicious node initiates attacks at the MAC layer and network layer simultaneously to make itself a node on the routing path by using a small CW and sending a fake routing message with small hop count. We observe that if a node initiates attacks its trust value will decrease obviously (less than 0.8). The behavior of back-off time manipulation of the malicious node will affect its idle time trust value, number of retransmissions trust value and physical layer trust value, so its trustworthiness will decrease to about 0.78. The selective forwarding attack will reduce the packet forward trust value of the malicious node. In the scenario of cross-layer attack, the parameters of both MAC layer and network layer will be affected, so the trust value of the malicious node will decrease markedly. The sinkhole attack will affect the hop count trust value, packet forward trust value, physical trust value of the malicious node because the malicious node will drop the Route Request (RREQ) packet, send the RREP packet with small hop count. Actually, the trust value of the malicious node is closely related to the selection of the attack parameters. In the cross-layer attack, in order to conceive its attack behavior, the malicious node will reduce the attack strength at the MAC layer and network layer, so in the simulation, the trust value of the malicious node in the cross-layer attack is slightly higher than that in the sinkhole attack.

**Figure 5:**
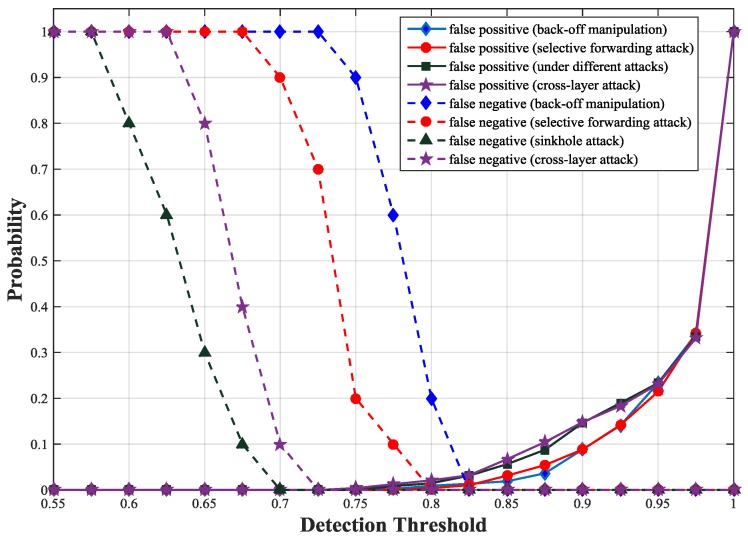
False positive and negative probabilities with different thresholds using simulation.

**Figure 6:**
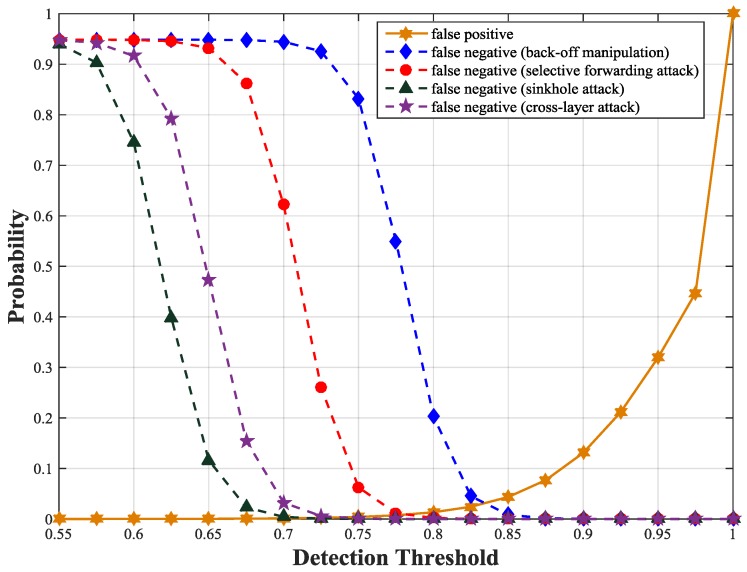
False positive and negative probabilities with different thresholds using formula calculation.

To get the detection threshold, we simulate false positive and false negative probabilities of our scheme with different thresholds under different types of attacks. We observe that the false positive probability curves under different attacks are similar except fluctuations within a small range. This is because the attacks have little influence on the trust values of unmalicious nodes but greater impact on those of malicious nodes. The intersection of false positive probability curve and false negative probability curve is the optimal trust threshold. Under the four attacks, we obtain an optimal detection threshold at which both false negative and false positive probabilities are minimized. As illustrated in Figure 5, the optimal detection threshold is about 0.83 at which both false positive and false negative probabilities are less than 0.05 for all types of attacks. We also obtain the false positive and negative probabilities according to Equations (22) and (23). Figure 6 shows the theoretical results are consistent with the simulation results.

**Figure 7:**
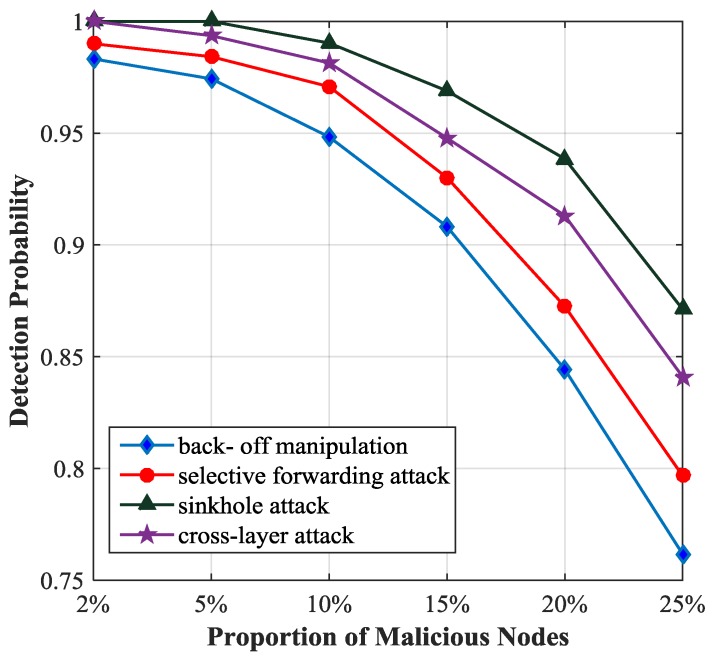
Detection probability with different proportion of malicious nodes.

We analyze the influence of the proportion of malicious nodes on the detection probability using the optimal threshold 0.83, as illustrated in Figure 7. We observe that the detection probability of sinkhole attack is the highest among the four types of attacks and the detection probability of back-off manipulation attack is the lowest because in the simulation sinkhole attack influences the trust value of the malicious node strongly and back-off manipulation attack has the minimal impact on it. If the proportion of malicious nodes is less than 5%, the detection probability will be more than 97%. If the proportion of malicious nodes is greater than 5%, the detection probability will decrease obviously, because with the increase of the number of malicious nodes, the trust value of the unmalicious node is closer to that of the malicious node, and then it is difficult to distinguish between the unmalicious node and the malicious node.

**Figure 8:**
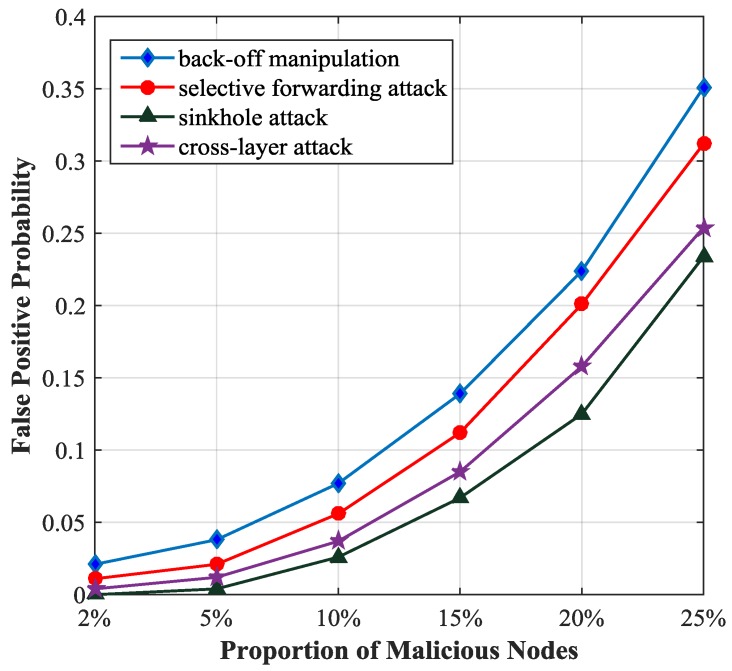
False positive probability with different proportion of malicious nodes.

Figure 8 describes the relationship between the false positive probability and the proportion of malicious nodes. If the proportion of malicious nodes is less than 5%, the false positive probability will be less than 0.05. It increases rapidly with the increase of the proportion of malicious nodes.

**Figure 9:**
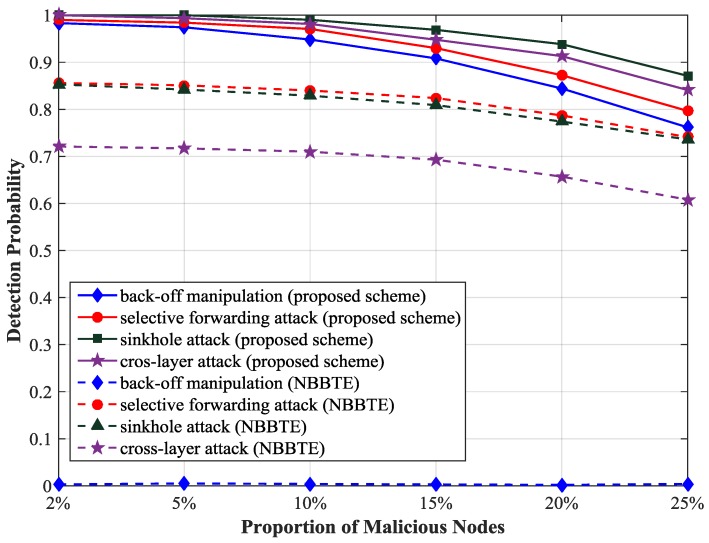
Comparison of the detection probability. NBBTE = Node Behavioral strategies Banding belief theory of the Trust Evaluation algorithm.

We compare the detection probability of our scheme with that of the NBBTE [23]. As shown in Figure 9, the detection probability of the selective forwarding attack and sinkhole attack have been improved by more than 10% and that of cross-layer attack has been improved by more than 20% as the proportion of malicious nodes is 2%, because many key parameters of multiple protocol layers are monitored and the trust values of SNs are calculated more accurately in our scheme. In NBBTE, the back-off manipulation attack can hardly be detected, because NBBTE only focuses on the node behaviors at the network layer, but ignores the malicious behaviors at the MAC layer, so it is not effective to detect the attacks at the MAC layer.

**Figure 10:**
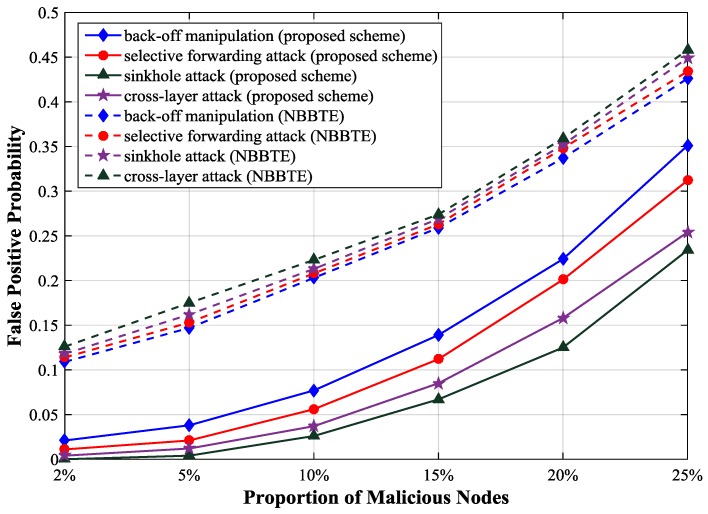
Comparison of the false positive probability.

Figure 10 shows that the false positive probability of NBBTE is higher than that of our scheme. Because we use the deviations of protocol parameters instead of the variations of node behaviors as NBBTE does for detecting malicious node, the reduction of the trust value caused by the normal change of the network can be avoided in our scheme. In NBBTE, the false positive probability curves under different attacks are very similar because the malicious behaviors have little influence on the trust values of normal nodes according to their algorithm.

### 4.3. Analysis of Communication Overhead

In our scheme, each sensor node monitors the key parameters of its neighboring nodes at each protocol layer and transmits the trust values toward the monitored nodes to its CH, so the communication overhead of our scheme mainly comes from the packets transmitted from SNs to the CH. As a result, the communication overhead of our scheme is related to the hop count from SNs to the CH. In NBBTE, it includes direct evaluation and indirect evaluation. In the direct evaluation, the monitoring node collects the key parameters of the monitored node and calculates the trust factors of the corresponding parameters. There is a factor of availability which evaluates the availability of the neighboring nodes. To obtain this factor, the monitoring node needs to transmit a HELLO packet and its neighboring nodes should reply to it with ACK-HELLO packets. In the indirect evaluation, the neighboring nodes of the monitored node will transmit their trust evaluation results towards the monitored node to the monitoring node as the indirect recommendation values. Thus, the communication overhead of NBBTE includes two parts, the HELLO packets for the factor of availability and the indirect trust evaluation from the recommendation nodes, which are related to the average number of neighboring nodes in the network.

In our scheme, all SNs transmit their evaluation results to their CH once in an observation period. Assuming there are n SNs in a cluster and the average hop count to the CH is $N_{h}$, the communication overhead of our scheme, $CO_{p}$ in an observation period can be expressed as $CO_{p}=nN_{h}$. As for the NBBTE, all SNs broadcast HELLO packets and reply to the HELLO packets of their neighboring nodes once in an observation period. Meanwhile, the neighboring nodes of each SN will send their recommendation values once in an observation period. Assuming the number of SNs is n, and the average number of neighboring nodes of a SN is $N_{a}$, the communication overhead of NBBTE, $CO_{N}$ in an observation period can be denoted by $CO_{N}=n\left(2N_{a}+1\right)$. We then analyze the communication overhead of the two schemes quantitatively in a network with 50 SNs and 1 CH under the circumstance that the two schemes have the same observation period Δt.

**Figure 11:**
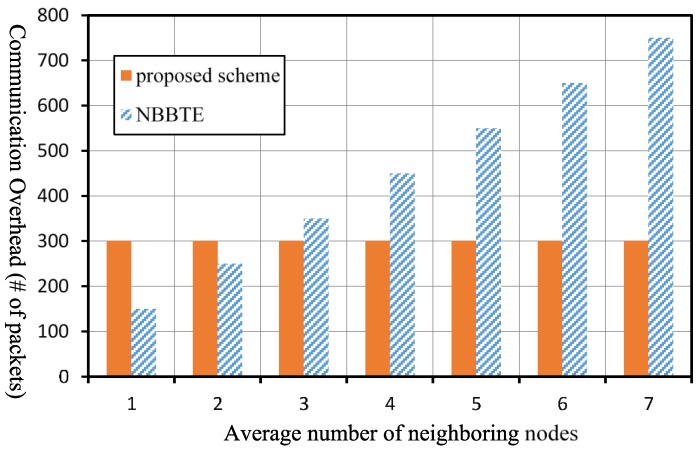
Comparison of the communication overhead under different number of neighboring nodes.

Figure 11 shows the comparison of the communication overhead of the two schemes under different number of neighbor nodes in the case that the average hop count $N_{h}$ in the network is 6. The communication overhead of the proposed scheme is not related to the number of neighboring nodes. If the average number of neighboring nodes is less than or equal to 2, the communication overhead of the NBBTE is less than that of our scheme. However, if the average number of neighboring nodes is greater than or equal to 3, the communication overhead of the NBBTE will be greater than that of our scheme.

**Figure 12:**
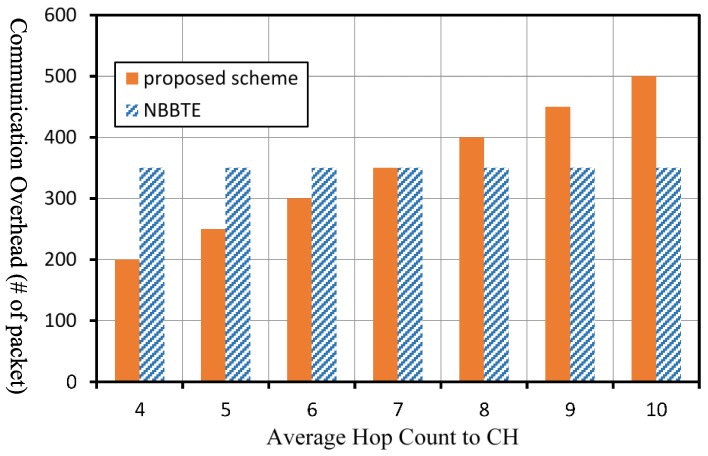
Comparison of the communication overhead under different average of hop count to the CH.

Figure 12 shows the comparison of the communication overhead of the two schemes under different average of hop count in the case that the average number of neighboring nodes $N_{a}$ in the network is 3. The communication overhead of our scheme will increase with increasing of the average hop count to the CH. If the average hop count to the CH is greater than 7, the communication overhead of our scheme will be greater than that of the NBBTE.

As described above, if the number of average neighboring nodes in a network is relatively large, the communication overhead of the NBBTE is greater than that of our scheme. If the hop count to the CH is relatively large, the communication overhead of our scheme is greater than that of the NBBTE. As a result, our scheme is more applicable to the network with less hop count. Moreover, from the angle of computation complexity, the calculation of trust value and the decision approach in the NBBTE are more complex than those in our scheme.

## 5. Conclusions

Wireless sensor networks are vulnerable to variety of attacks at different protocol layers. In the existing trust-based intrusion detection schemes, there is no unified standard to select trust factors, and cross-layer attacks are seldom considered. In order to identify malicious nodes more efficiently, we have proposed a protocol layer trust-based intrusion detection scheme for WSNs. In our scheme, the key parameters of different protocol layers are monitored and the trust values of sensor nodes can be calculated according to the deviations of parameters. By comparing the trust value with a predefined threshold, we can decide whether the sensor node is compromised or not. It can describe the trust values of sensor nodes more accurately by considering the deviations of parameters of multiple layers, hence our proposed scheme is effective for detecting cross-layer attacks. We utilized the t-distribution and simulation to analyze the detection probability and false positive probability of our scheme. The results indicate that there exists an optimal trust threshold at which both false positive and false negative probabilities are minimized. Our proposed scheme outperforms the NBBTE scheme in terms of the detection probability and false positive probability. The weakness of our scheme is the communication overhead will increase with the increasing of the hop count to the CH. Our scheme is extendable, the selection of the trust factors at different protocol layers can be adjusted according to the requirements of a system, and it is applicable to both clustered WSNs and flat WSNs. As for future works, we will analyze the attacks initiated at the transport layer and application layer, as well as MAC-Transport cross-layer attack, Network-Application cross-layer attack and their influence on the protocol parameters to further optimize our scheme. In addition, we will perform experiments to test the performance of our scheme on a real WSN testbed to assess its real-life performance.

## Figures and Tables

**Table 1:** Assumed values of system parameters. MAC = medium access control, AODV = Ad-hoc On-Demand Vector.

Parameter	Value	Parameter	Value
Size of network	100 m × 100 m	w_1_, w_2_, w_3_	1/3
Number of SNs	49	Number of CH	1
Trust update interval	10–100 min	p_1_, p_2_, q_1_, q_2_	1/2
MAC protocol	802.11 DCF	Routing protocol	AODV
t_DIFS_	50 μs	t_SIFS_	10 μs
Slot time	20 μs	t_ACK_	112 μs
Size of Route Request (RREQ) packet	176 bits	Size of Route Reply(RREP) packet	176 bits
